# Effect of Amaranth-Containing Dietary Intervention in Improving Hemoglobin Concentration: A Systematic Review and Meta-Analysis

**DOI:** 10.3389/phrs.2024.1607597

**Published:** 2025-01-03

**Authors:** Mekdes Tigistu Yilma, Aberash Eifa, Mehretu Belayneh, Alemselam Zebdewos Orsango

**Affiliations:** ^1^ College of Medical and Health Sciences, Wollega University, Nekemte, Ethiopia; ^2^ Faculty of Health Sciences, Hawassa University, Hawassa, Ethiopia

**Keywords:** amaranthus, hemoglobin, undernutrition, intervention, systematic review

## Abstract

**Objective:**

Amaranth, a nutritious iron source, is known for treating anemia in young children and lactating mothers, but its effectiveness in reducing hemoglobin concentration needs further investigation. Therefore, this study aimed to summarize the effectiveness of amaranth-based food interventions in improving hemoglobin concentration.

**Method:**

A randomized controlled trial and quasi-experimental study conducted since 2000 were searched in databases like PubMed, Scopus, Embase, Cochrane, AJOL, and Web of Science using prespecified keywords. Excel and Stata 17 were used for data extraction and analysis. Methodological quality was assessed using the JBI systematic review critical appraisal tool. Meta-analysis was done to estimate the overall intervention effect.

**Result:**

Ten studies were included from 1,032 articles (n = 1,225). The standardized mean hemoglobin concentration difference between groups was positive, with an overall effect of 0.08 (95%CI: −0.11, 0.26; p = 0.433), where I^2^ is 57.1%.

**Conclusion:**

The studies’ interventions showed positive effects on hemoglobin concentration, but their effectiveness was not statistically significant. This suggests the need for research on the impact of different cooking methods on iron bioavailability, phytic iron ratio, and intervention effects across different populations.

**Systematic Review Registration:**

Identifier PROSPERO CRD42023476402.

## Introduction

Globally, the prevalence of anemia has been decreasing; however, it remains a public health concern, particularly among young children 40% (36%–44%) and pregnant women 36% (34%–39%) [1]. Anemia can result in various adverse outcomes, including adverse perinatal and birth outcomes, such as increased risk of postpartum hemorrhage, sepsis, growth and developmental issues in children, and even death in severe cases [2–4]. To reduce the burden of nutritional deficiency anemia, which is regarded as one of the top causes of anemia, various interventions have been implemented, such as iron supplementation, fortification and dietary interventions [5]. Among these dietary interventions, amaranth is recognized for its therapeutic effect on the treatment of anemia in young children and lactating mothers [6].

Amaranth, also known as *Pseudocereal*, which belongs to the genus *Amaranthus*, is an ancient crop with a rich history and cultivation in various parts of the world, and it is believed to have originated in South America [7]. There are various types of amaranth species, like *Amaranthus caudatus L., A. cruentus L.,* and *A. hypochondriacus Leucocarpus*. These species were cultivated in different parts of America, including Mexico, Guatemala, and the Andrea region, as well as Asia and Africa [7–9]. Despite being discouraged for its use in ancient religious ceremonies, amaranth holds special religious significance in some regions of countries like India and Pakistan [10]. A few decades ago, its cultivation and utilization increased following the emergence of evidence regarding its nutritional value [11].

The production of grain amaranth, with a tiny seed less than 1 mm in diameter, outperforms corn in terms of yield per unit of land [7]. Its ability to thrive with efficient water use and adaptability makes it a crucial crop in the global food system by addressing the challenges of climate change and population growth. Besides the grains, amaranth’s leaves are edible and have spinach-like tastes [10, 12], though their chemical compositions differ from spinach. On average, amaranth comprises 61.3–76.5 g of crude carbohydrate, 13.1–21.5 crude protein (rich with lysine), 5.6–10.9 g crude fat and 2.7–5 g fiber per 100 g of amaranth. It also offers a balanced amino acid composition [12] and provides 7.61 mg of iron per 100 g of amaranth [13]. Moreover, amaranth leaves also provide 27.3 mg/100 g iron [14], which equals seven times more iron content than lettuce [15].

With its adaptability and nutritional composition, amaranth is a promising crop for combating malnutrition and food insecurity across the globe [11]. Amaranth can be combined with other cereals to produce different food products [10, 12], and its higher oil content makes it an alternative seed for different industries like oil production, pharmaceutics and cosmetics [16, 17]. The oxidative stability of the amaranth oil is even higher than that of commonly used sunflower oil. The oil content of raw *A. caudatus L.* and *A. cruentus L.* were 7.1% and 8.5%, with a high triacylglycerol (80.3%–82.3%) content [17]. Amaranth oil has been evidenced to have beneficial effects for patients with coronary heart disease and hypertension through reduction of total cholesterol, low-density lipoprotein and very low-density lipoprotein [18]. Such positive effects of this nutritious grain were also evidenced in its significant nutritional value [19–24].

Despite the abundance of literature suggesting the nutritional and therapeutic benefits of amaranth, its practical utilization for alleviating nutritional anemia demands further attention. This is highlighted by Anemia remaining a significant public health concern in key producers of amaranth, such as China, Kenya and several sub-Saharan African countries, where anemia ranges from 11% to 60% [8, 25–27]. The prevalence of nutritional anemia, even in amaranth-producing countries, raises questions about consumption patterns and the effectiveness of amaranth in reducing anemia. This underscores the limitation of evidence on the effectiveness of amaranth-containing foods on hemoglobin concentration. Additionally, while there are no previously published systematic reviews and meta-analysis to our knowledge on this specific topic, pre-identified empirical primary studies have shown opposing conclusions on the phenomenon [28, 29]. Therefore, this systematic review and meta-analysis aims to summarize the effectiveness of amaranth-based food products in improving hemoglobin concentration.

## Methods

### Study Desing

A systematic review and meta-analysis of primary studies was conducted in accordance with the Preferred Reporting Items for Systematic and Meta-Analysis-2020 (PRISMA-2020) guideline [30], as shown in Supplementary Material S1. The study also followed a pre-designed systematic review protocol that was registered in the International Prospective Register of Systematic Reviews (PROSPERO)—registration number CRD42023476402. Following the preliminary search, the scope of the review became narrowed to determining the effects of amaranth-containing food products on hemoglobin concentration.

### Eligibility Criteria

As we aimed to assess the best available evidence related to the effectiveness of amaranth, interventional studies were targeted to be included in the review. Accordingly, a Randomized Controlled Trial (RCT), Quasi-Experimental Study (QE) and pre-post observational studies aimed to assess the effectiveness of amaranth-based food intervention on individuals aged 6 months or older regardless of their health condition were included in this review. To minimize bias in selecting studies [31], both published and unpublished studies available online from January 2000 to April 2024 were targeted in a comprehensive database search.

Additional eligibility criteria included population, intervention types, comparator and outcome. Amaranth-containing food can be consumed by different age groups, including young children and adults [22, 32]. Children older than 6 months should start complementary food [33]. Therefore, the population in this study consists of individuals over 6 months of age, regardless of their health status or any demographic characteristics. In terms of intervention type, studies involving food interventions that included amaranth as a key ingredient, regardless of specific food type, were targeted. The intervention could have been implemented in different settings like households, schools, and communities. Comparisons were made either against other functional foods or in the absence of any interventions for the control group.

The main outcome of this study was to determine the effect of amaranth on anemia, which is assessed by hemoglobin levels. Anemia’s diagnosis and severity are determined by the cut-off value of hemoglobin concentration, which is influenced by factors like age, sex, health status, and altitude [34]. Hence, studies that assessed the levels of hemoglobin in both the intervention and control groups were sought by measuring the mean and standard deviation of hemoglobin before and after the intervention. Then mean difference was generated to see the effect of the intervention on hemoglobin concentration, as recommended in the Cochrane Handbook [31].

In converse, studies assessing amaranth’s nutritive value, qualitative investigation, book chapters, compressive reviews, systematic reviews, studies that missed reporting hemoglobin before and after the intervention in both the intervention and control groups and studies published in another language other than English were excluded from the review.

### Databases, Search Strategies and Study Selection

Online databases like PubMed, Scopus, Cochrane, Embase, Google Scholar, AJOL, and Web of Science were searched for the records. Furthermore, ProQuest’s dissertation and gray literature from search engines like Google were used to search unpublished studies. The initial search was done compressively using the following keywords: “effectiveness,” “efficacy,” “amaranth,” “amaranthus,” “amaranthus cruentus OR amaranthus caudatus OR amaranthus hypochondriacus,” “food OR snack,” and “intervention.” Then the search was updated by adding keywords like hemoglobin OR Anemia OR nutrition and by indexing dated between 1 January 2000 and 13 April 2024. The reference lists of eligible studies were also evaluated for potential articles based on selection criteria. The database search strategies used are provided as a supplementary document (Supplementary Material S2).

The bibliographies of articles identified from all databases were recorded and exported to EndNote [35] and stored in a respective database labelled group. MY and AE used the Covidence Systematic Review online software^1^ to independently review and screen articles by title, abstract and full text based on eligibility criteria. Any discrepancy was resolved by the third reviewer, AZ, and through discussion.

### Methodological Quality

Methodological quality was assessed using the JBI systematic review critical appraisal tool for systematic review of effectiveness, which has a separate format to examine the quality of RCT and quasi-experimental studies [36]. To ensure the consistency of the data, the assessment was done independently by MY and AE using a table. In a situation where the design of the study was not clear, we decided on the protocol of the study, particularly based on randomization and the presence of a control group. Any discrepancy was resolved by discussing and reviewing the article together.

### Data Collection, Analysis and Synthesis

Data were extracted from eligible studies in Excel using a pre-structure sheet by two independent reviewers (MY and AE) focusing on study design, year of publication, type of study, sample size, type of food intervention (drink, meal, snack, juice or else), part of amaranth used (leaves or grain), amaranth species, population, comparator food, follow up time, frequency of meal, statistical analysis, findings and conclusion. Any discrepancy was resolved by discussing and extracting the data together.

The characteristics of included studies, like the type of intervention, target population, the health status of the study participants, species, used amaranth part (leaves or grain), frequency of consumption and follow-up period, were synthesized and described in the table. Unreported standard deviation was obtained using the square root of *n* times the width of the confidence interval divided by 3.92 [29].

Meta-analysis was conducted using Stata version 17 to generate the pooled effect of amaranth-containing food on hemoglobin concentration. The standardized mean difference (Hedges’ g) was used to estimate the overall effect size considering variation in the population, intervention, and measurement of outcome. The pooled estimate was summarized and presented in a forest plot. A random effect model was used considering the characteristics of studies and observed heterogeneity. Publication bias was assessed using a funnel plot and Egger’s test. In response to a significant small study effect, trim-and-fill estimation was done and described using a trim-and-fill funnel plot [37]. A subgroup analysis was done to examine the source of heterogeneity.

### Certainty of Evidence

Certainty of evidence and summary of findings on the effectiveness of amaranth-containing food on hemoglobin concentration was assessed using a Grading of Recommendations, Assessment, Development and Evaluation (GRADE) approach and the summary of findings table is presented as a supplementary document (Supplementary Material S3).

## Result

### Search Results and Study Selection

**FIGURE 1:**
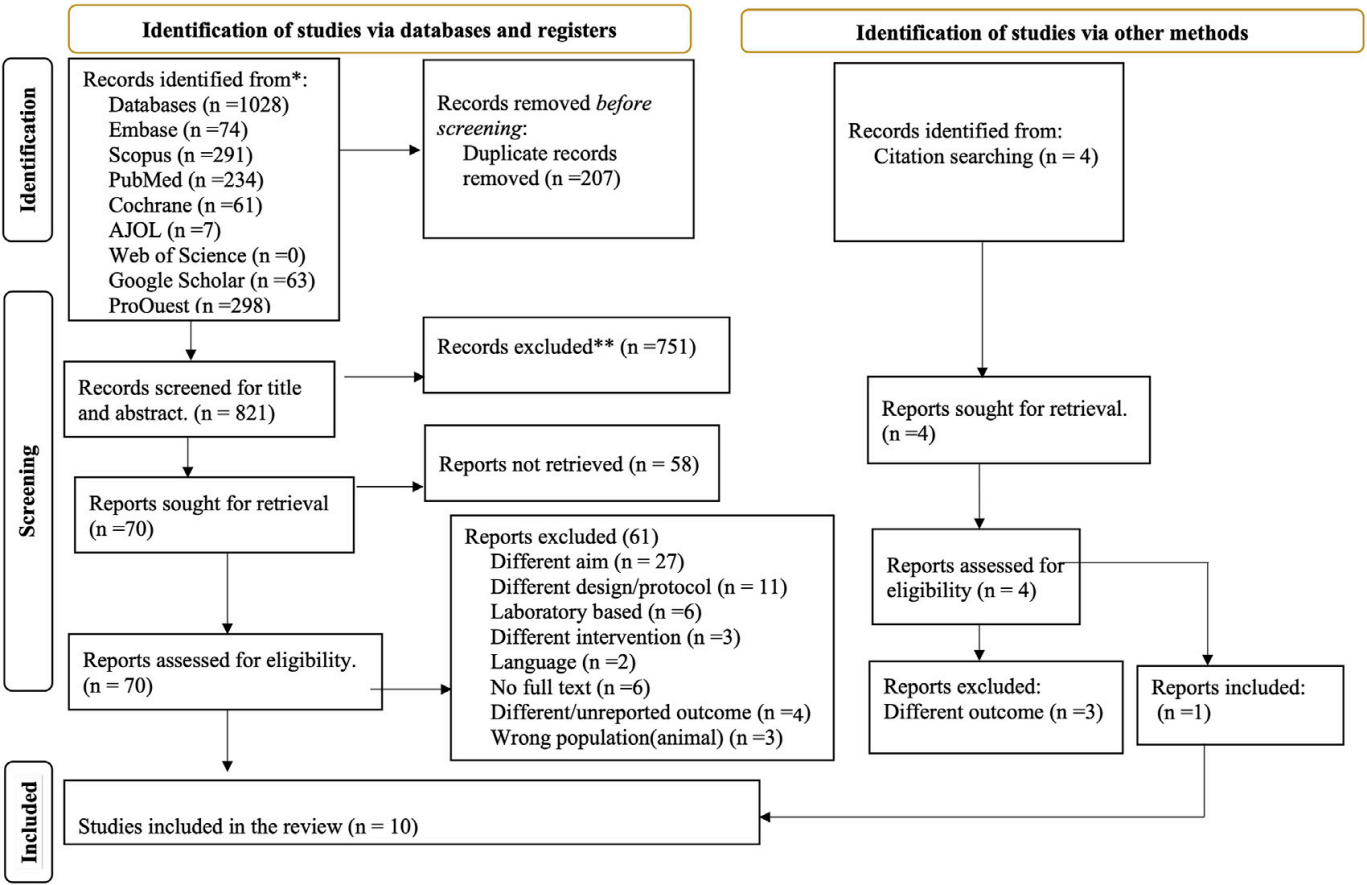
Preferred reporting items for systematic review and meta-analysis: the PRISMA flow diagram of screened, evaluated and included amaranth containing dietary intervention studies (Global systematic review, 2000–2024).

A systematic search of studies was conducted to access published and unpublished studies from databases like Embase, Scopus, PubMed, Cochrane and AJOL. Furthermore, Google Scholar and ProQuest were used for articles and dissertations. A total of 1,032 articles were found from database and citation search, and only 10 articles were found to fulfill the inclusion criteria from 70 overall records screened in full text, as depicted in Figure 1.

### Methodological Quality of Included Studies

Methodological quality was assessed using the JBI systematic review critical appraisal tool for a systematic review of effectiveness review of RCT and QE studies [36]. The baseline characteristics of comparison groups in some of the QE studies were different in some of the included quasi-experiment study designs [38, 39]. The study design was not clearly specified as RCT or quasi-experimental [28, 39]. As a result, we considered the study design to be quasi-experimental design based on the study protocol. One QE study did not discuss the treatment other than the intervention given for the comparison group. Furthermore, the statistical analysis of the included studies was not appropriate to compare differences between groups (Table 1) [28].

**TABLE 1:** Critical appraisal results for the included amaranth containing dietary intervention quasi-experimental studies (Global systematic review, 2000–2024).

Studies	Critical appraisal questions									Score
	Q1	Q2	Q3	Q4	Q5	Q6	Q7	Q8	Q9	
Ginting et al. [38]	Y	UC	Y	Y	Y	Y	Y	UC	Y	7
Nawiri et al. [39]	Y	UC	Y	Y	Y	UC	Y	Y	Y	7
Singh et al. [28]	Y	Y	UC	Y	Y	Y	Y	N	N	6
Muliani et al. [40]	Y	Y	Y	Y	Y	UC	Y	UC	Y	7
Total % of positive scores	100	50	75	100	100	50	100	25	75	

JBI critical appraisal checklist for quasi-experimental studies (Y, yes; N, No; UC, Unclear; N/A, Not Applicable).

Q1, Is it clear in the study what is the “cause” and what is the “effect” (i.e., there is no confusion about which variable comes first)?

Q2, Were the participants included in any comparisons similar?

Q3, Were the participants included in any comparisons receiving similar treatment/care, other than the exposure or intervention of interest?

Q4, Was there a control group?

Q5, Were there multiple measurements of the outcome both pre and post the intervention/exposure?

Q6, Was follow up complete and if not, were differences between groups in terms of their follow up adequately described and analyzed?

Q7, Were the outcomes of participants included in any comparisons measured in the same way?

Q8, Were outcomes measured in a reliable way?

Q9, Was appropriate statistical analysis used?

The assessment of the methodological quality of RCT showed that allocation concealment was only described by half of the included studies. Most studies did not report information regarding the blinding of the study of participants, treatment providers and outcome assessors [41–43]. One study discussed that all measurements were blind. However, the authors did not describe blinding participants, those delivering treatment and outcome assessors [44]. The baseline characteristics of study participants were also different in some of the studies [32, 42]. Regarding the statistical analysis, some studies did not report the approach they followed as an intention-to-treat or per protocol (Table 2) [41, 43, 45, 46]

**TABLE 2:** Critical appraisal results for included amaranth containing dietary intervention randomized controlled trial studies (Global systematic review, 2000–2024).

Studies	Appraisal questions													Score
	Q1	Q2	Q3	Q4	Q5	Q6	Q7	Q8	Q9	Q10	Q11	Q12	Q13	
Fitriani et al. [43]	Y	UC	Y	UC	UC	Y	Y	Y	UC	Y	Y	Y	N	8
Konyole et al. [29]	Y	Y	Y	Y	Y	Y	Y	Y	Y	Y	Y	Y	Y	13
Macharia et al. [42]	Y	UC	N	UC	UC	UC	Y	Y	Y	Y	Y	Y	Y	8
Orsango et al. [32]	Y	Y	N	Y	Y	Y	Y	Y	Y	Y	Y	Y	Y	12
Stiller et al. [41]	Y	UC	Y	UC	N	UC	UC	Y	UC	Y	Y	N	Y	6
Hoevenet al. [44]	Y	Y	N	UC	UC	UC	Y	Y	Y	Y	Y	Y	Y	9
Total % of positive scores	100	50	50	33.3	33.3	50	83.3	100	66.7	100	100	83.3	83.3	

JBI critical appraisal checklist for randomised controlled trials (Y, yes; N, No; UC, Unclear; N/A, Not Applicable).

Q1, Was true randomization used for assignment of participants to treatment groups?

Q2, Was allocation to treatment groups concealed?

Q3, Were treatment groups similar at the baseline?

Q4, Were participants blind to treatment assignment?

Q5, Were those delivering treatment blind to treatment assignment?

Q6, Were outcomes assessors blind to treatment assignment?

Q7, Were treatments groups treated identically other than the intervention of interest?

Q8, Was follow up complete and if not, were differences between groups in terms of their follow up adequately described and analyzed?

Q9, Were participants analyzed in the groups to which they were randomized?

Q10, Were outcomes measured in the same way for treatment groups?

Q11, Were outcomes measured in a reliable way?

Q12, Was appropriate statistical analysis used?

Q13, Was the trial design appropriate, and any deviations from the standard RCT design (individual randomization, parallel groups) accounted for in the conduct and analysis of the trial.

### Characteristics of Included Studies

A total of ten studies were included, of which 60% them were RCT and 40% were QE studies. Most studies were published between 2012 and 2019 from five countries which are Indonesia [3], Kenya [3], South Africa [1], Ethiopia [1] and India (one published and one unpublished). About 30% of included studies were community based and 20% were school based. Most of the studies (70%) included a younger population, and one study included post-partum women without age specification. The total sample size was 1,225 (614 in the intervention and 611 in the control group), ranging from 20 to 334 and follow-up period from 2 to 72 weeks. Furthermore, about 70% of included studies used leaves of amaranths to prepare the intervention like juice, herbal drinks, porridge and bread (Table 3).

**TABLE 3:** Characteristics of included amaranth containing dietary intervention studies (Global systematic review, 2000–2024).

Study	S year	Study type	P year	Country	Design	Tn	In	Cn	Age	Health status	Follow (week)
Fitriani et al. [43]	2018	Published	2020	Indonesia	SRCT	128	64	64	18–60 years	Anaemic	4
Ginting et al. [38]	2019	Published	2021	Indonesia	QE	30	15	15	20–40 years	Healthy	2
Konyole et al. [29]	2013	Published	2019	Kenya	PRCT	334	167	167	6 months	Healthy	36
Macharia-Mutie et al. [42]	2011	Published	2012	Kenya	PRCT	186	93	93	1–5 years	Healthy	16
Nawiri et al. [39]	NR	Published	2013	Kenya	QE	107	56	51	2.5–6 years	Healthy	13
Orsango et al. [32]	2017	Published	2020	Ethiopia	CRCT	100	50	50	2–5 years	Anaemic	24
Singh et al. [28]	NR	Published	2023	India	QE	20	10	10	5–11 years	Anaemic	6
Stiller et al. [41]	2016	Unpublished	2020	India	CRCT	123	58	65	6–39 months	Anaemic	72
Faber et al. [44]	2012	Published	2015	South Africa	PRCT	167	86	81	6–12 years	Healthy	12
Muliani et al. [40]	UC	Published	2017	Indonesia	QE	30	15	15	.NR	Post-partum women	2

Study	Species	Used	Prepared food	Comparator	Consumption/week	Measurement	Conclusion
Fitriani et al. [43]	*A. tricolor*	leaves	Herbal drink	Iron supplement	10	NR	No significant difference
Ginting et al. [38]	*A.tricolor*	leaves	Juice (amaranth alone)	Beta Vulgaris juice	5	NR	Increase
Konyole et al. [29]	*A.cruentus*	grain	Complementary	CSB+	NR	NR	Decrease
Macharia-Mutie et al., 2012	not specified	grain	Porridge	Plain maize porridge	.NR	Sysmex hematological analyzer (KX-21, Sysmex)	No significant difference
Nawiri et al. [39]	not specified	leaves	Food	White cabbage	5	hemocue analyzer (Model B-Hemoglobin, Angelholm, Sweden)	Increase
Orsango et al. [32]	not specified	grain	Bread	Maize bread	7	HemoCue analyser 301 (Angelholm, Sweden)	Increase
Singh et al. [28]	not specified	leaves	Balls	Nothing	14	Haemoglobinometer (Fully Cell-Counter Automatic)	Increase
Stiller et al. [41]	*A. tricolor*	leaves	Cooked food	Nothing	2	HemoCue201+	No significant difference
Faber et al. [44]	*A.cruentus*	leaves	Cooked food	School meal	5	Coulter^®^ Ac·T™ 5diff CP	No significant difference
Muliani et al. [40]	*A. tricolor*	leaves	Red spinach extract tablet (amaranth alone)	Iron capsule	21	NR	Increase

S Year: NR is for study year not reported, UC: unclear; Design: SRCT: Standard RCT, QE: quasi experimental, PRCT: Parallel RCT, and CRCT: Cluster RCT; Tn: Total sample size; In: sample size for intervention group; Cn: Sample size for control group: CSB+: fortified corn–soy blend plus.

### Effectiveness of Amaranth-Containing Food on Improving Hemoglobin Level

The minimum standardized mean difference for the effect of amaranth-containing food on hemoglobin was zero (95%CI: −0.29, 0.29) [42] and the largest effect size was 1.46 (0.51, 2.41) [28]. The forest plot indicates the presence of heterogeneity where I^2^ is 57.1%. To investigate the possible reason behind observed variation, subgroup analysis was conducted, and it showed that there is a higher heterogeneity among studies that recruited anemic subjects (86.02%) than healthy subjects (no heterogeneity). Similarly, heterogeneity was higher among quasi-experimental studies (75.3%) than RCT (36.3%). Studies that used amaranth leaves showed a higher heterogeneity (71.7%) than those that used amaranth grain (67.3%).

**FIGURE 2:**
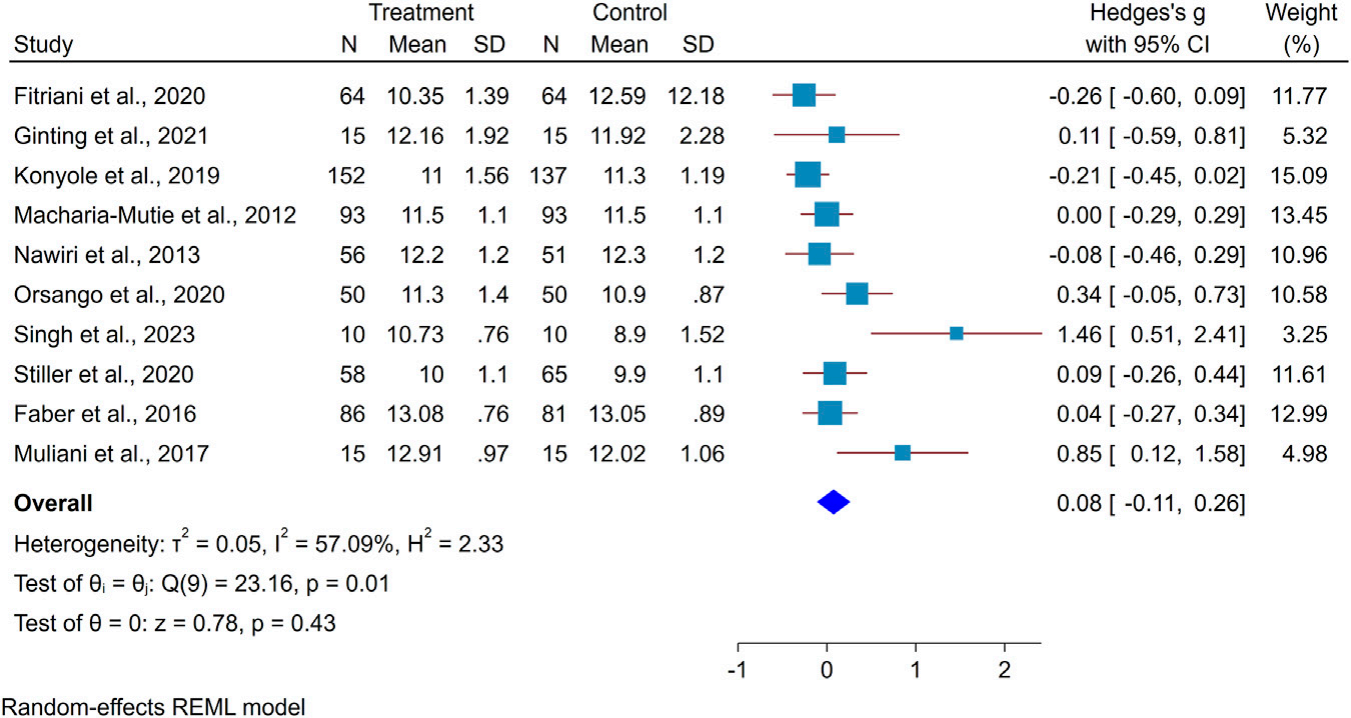
Forest plot showing the effect of amaranth containing dietary intervention on hemoglobin level (Global systematic review and metanalysis, 2000–2024).

The standardize mean hemoglobin concentration difference between the intervention and control groups found to be positive, with the overall pooled effect of amaranth-containing food on hemoglobin concentration being 0.08 (95% CI: −0.11, 0.26). The statistical test of the overall effect size is not different from zero (p-value 0.4330). The studies by Singh et al. [28] and Muliani et al. [40] may affected the findings with a low sample size and large effect size (Figure 2). To identify the most influential studies, a leave-one-out graph was constructed, and it was found that omitting Muliani et al. [40] and Singh et al. [28] decreased the overall effect size by 0.07.

### Assessment of Bias

The asymmetric distribution of the study in the funnel plot indicated the presence of publication bias (Supplementary Material S4). Furthermore, egger’s test of small study effect (p-value < 0.001) suggests the presence of a small study effect. The trim-and-fill method which used to correct publication bias by imputing two studies where only one falls within alpha < 0.05. The trim-and-fill estimator changes the direction and magnitude of the overall effect size −0.01 (95%CI: −0.32, 0.3) (Supplementary Material S4).

### Subgroup Analysis

**FIGURE 3:**
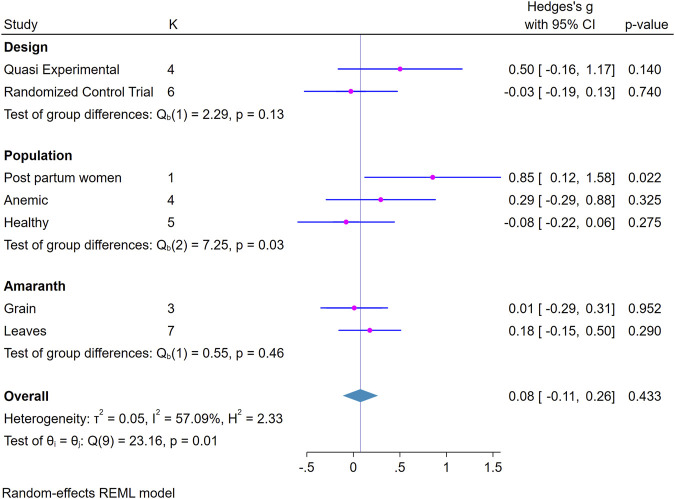
Forest plot showing subgroup analysis of the effect of amaranth containing dietary intervention on hemoglobin level (Global systematic review and metanalysis, 2000–2024).

The effect of the intervention was compared across different groups such as study design, population and amaranth part used. Accordingly, the result showed a significant positive effect of the intervention for postpartum women, 0.85 (95%CI; 0.12, 1.58) (Figure 3).

## Discussion

In this systematic review and meta-analysis, we summarized the effectiveness of amaranth-containing food interventions on the concentration of hemoglobin as reported in primary studies. The pooled standardized mean difference showed that the interventions were not found to have a significant effect on hemoglobin concentration. However, it was found to have a significant effect on postpartum women. This might be due to post-partum women having a 20%–37% risk of iron deficiency anemia during pregnancy could start to return back to the pre-pregnancy state parallel to the physiological changes during postpartum [47]. On the other hand, although factors like failure to exclusive breastfeeding and multiparity were known to be associated with the occurrence of anemia [48], which could be partly mediated by the preexisting physiologic anemia during pregnancy and childbirth due to enhanced demands and blood loss [49]; the provision of amaranth-containing foods could serve as an important source of nutritional intervention to alleviate nutritional anemia for postpartum women. The effectiveness of such interventions could be influenced by factors such as the target population, the type of food prepared, the method of preparation, the frequency of consumption, and the inclusion of deworming.

To ultimately utilize the high content of iron in amaranth, avoidance of the factors that inhibit iron absorption, like deworming, and reducing intake of caffeine-containing drinks like coffee and tea are indispensable [50]. However, only three included studies [32, 38, 39] reported considering these factors during intervention provision. These studies found a significant positive effect of the intervention following providing anthelmintic medication to the participants prior to the intervention [32, 39] and provided clear instructions about food that should be avoided during the intervention period [38]. This underscores the importance of deworming for improved outcomes following nutritional intervention, as also suggested elsewhere [51]. Likewise, an amaranth-based nutritional intervention should prioritize managing the consumption of coffee and determine of phytic iron ratio. The failure to deworm and limit caffeine consumption in most of the reviewed studies [28, 40, 41, 43, 44, 52] could partly explain the lack of significance of amaranth-containing foods in enhancing hemoglobin concentration.

In addition to limiting previous consumption habits like restricting caffeine intake and deworming against intestinal parasites, which reduce iron absorption, the recipe preparation methods are also essential. Though amaranth is the best source of iron [13] and its inclusion in the diet improves the nutrient content of the food [53], the preparation method could affect the concentration of phytate and bioavailability of iron [54]. Different studies have reported methods of preparation of food that reduce the phytate content of amaranth, like soaking, germinating and fermentation [32, 55]. It is evidenced that boiling [54], popping and toasting [56] decrease iron, whereas soaking, germination [57], and fermentation [56] increase iron contents of amaranth. Moreover, germination decreases the concentration of phytic acid from amaranth [58]. On the contrary to the high content of iron, the presence of antinutrients like phytate could be the reason for the insignificant effect of amaranth-based food, as only two studies took measures of reducing phytate and supporting the micronutrient bioavailability [29, 32]. This might be due to the absorption of iron affected by the concentration of phytate [59]. The observed non-significant intervention effect could be linked to these preparation methods issues, highlighting the importance of taking these measures before preparing amaranth-containing food to maximize iron availability and absorption. Furthermore, various amaranth-containing foods were provided in combination with other functional grains or leaves, which could influence the bioavailability and absorption as their contents’ negative effects were not controlled.

This systematic review is the first to summarize the effect of amaranth-containing dietary intervention on improving hemoglobin concentration. The comprehensive and updated searches that ensure the inclusion of all relevant studies are among the strengths of this review. The main limitation of this review is the heterogeneity of included studies in terms of factors like the health status of the population, age variation, type of intervention, and frequency of consumption. Such inconsistency and the observed publication bias may underestimate the overall effect of the intervention. Therefore, further studies on amaranth-based dietary intervention in different contexts and target populations should be conducted. Furthermore, there was no clear information across the study about the ratio of amaranth in the prepared intervention. Which also affects the findings and conclusion of the review. Bias related to allocation concealment and blinding could also affect the treatment effect in either way. Therefore, an experimental study with the application of all the standard procedures is needed to assess the effect of amaranth-based dietary intervention. Another potential issue could be the loss of studies that might have been included if we had incorporated other languages. However, we acknowledge this limitation, considering that most databases index abstracts of studies written in non-English languages. Therefore, we believe the number of studies lost due to this limitation is minimal.

## Conclusion

Amaranth-containing foods could serve as a potential source of iron to help increase hemoglobin levels among postpartum women. However, for other groups, we didn’t find strong evidence supporting a significant increase in hemoglobin concentration following dietary intervention with amaranth. Despite its high iron content, amaranth-containing food interventions appeared to have little or no effect on improving hemoglobin concentrations. Factors such as the phytic-to-iron ratio in the food and preparation method may have affected the bioavailability of iron in primary studies. Therefore, future research should investigate the impact of different cooking methods on iron bioavailability and the phytic-to-iron ratio to standardize preparation methods and enhance the effectiveness of amaranth-containing food in raising hemoglobin concentrations. Recommendations for improving the effectiveness of amaranth-food consumption should include deworming, avoiding caffeine intake, and reducing phytate content through soaking, germination, and fermentation. Meanwhile, boiling, popping, and toasting should be avoided, as they reduce the iron content of amaranth-containing foods. This study also underscores the need for further investigation of the intervention effect in different populations.

## Footnotes

1
http://www.covidence.org/

## Abbreviations

RCT, Randomized Controlled Trial; QE, Quasi Experimental Study; PICOS, Population, Intervention, Comparison, Outcome and Study design.
