# Supplementary material for: Effect of Amaranth-Containing Dietary Intervention in Improving Hemoglobin Concentration: A Systematic Review and Meta-Analysis
Source: Public Health Rev. 2025 Jan 3;45:1607597. doi: 10.3389/phrs.2024.1607597 (PMC11738614; doi:10.3389/phrs.2024.1607597)
Supplement: Supplementary file 1 [file DataSheet4.docx]

Figure 4 A: funnel plot for publication bias at alpha equals to 1%, 5% and >10%; B: Trim fill funnel plot at alpha equals to 1%, 5% and >10%.
