# Supplementary material for: Effect of Amaranth-Containing Dietary Intervention in Improving Hemoglobin Concentration: A Systematic Review and Meta-Analysis
Source: Public Health Rev. 2025 Jan 3;45:1607597. doi: 10.3389/phrs.2024.1607597 (PMC11738614; doi:10.3389/phrs.2024.1607597)
Supplement: Supplementary file 3 [file DataSheet3.docx]

| **Summary of findings:** | | | | | | |
| --- | --- | --- | --- | --- | --- | --- |
| **Amaranth containing food compared to other nutrition intervention or no intervention in improving heamoglobin concentration** | | | | | | |
| **Patient or population:** individuals aged ≥ 6-month-old regardless of their health status  **Setting:** global  **Intervention:** Amaranth containing food  **Comparison:** other nutrition intervention or no intervention | | | | | | |
| Outcome № of participants (studies) | Relative effect (95% CI) | **Anticipated absolute effects (95% CI)** | | | Certainty | What happens |
|  |  |  |  | **Difference** |  |  |
| Hemoglobin level (Hemoglobin) follow-up: range 2 weeks to 72 weeks № of participants: 1225 (Six RCT and four QES) | - | - | - | SMD **0.08 SD higher** (-0.11 lower to 0.26 higher) | ⨁⨁⨁◯ Moderate^a,b,c^ | The high concentration of phytate and preparation method could affect the bioavailability of iron. Furthermore, there was no clear information across the study about the ratio of amaranth in the prepared intervention. This might affect the findings and conclusion of the review. |
| ***The risk in the intervention group** (and its 95% confidence interval) is based on the assumed risk in the comparison group and the **relative effect** of the intervention (and its 95% CI).  **CI:** confidence interval; **SMD:** standardised mean difference; **QES**: Quasi-experimental study | | | | | | |
| **GRADE Working Group grades of evidence** **High certainty:** we are very confident that the true effect lies close to that of the estimate of the effect. **Moderate certainty:** we are moderately confident in the effect estimate: the true effect is likely to be close to the estimate of the effect, but there is a possibility that it is substantially different. **Low certainty:** our confidence in the effect estimate is limited: the true effect may be substantially different from the estimate of the effect. **Very low certainty:** we have very little confidence in the effect estimate: the true effect is likely to be substantially different from the estimate of effect. | | | | | | |

#### Explanations

a. Inconsistencies may arise from inconsistent food type used as intervention, the type of amaranth used (leaves or seed), the other ingredients used along with amaranth, the preparation method and the difference in the targeted population.

b. Considering the size and 95% confidence interval which overlaps no effect or does not exclude no effect the evidence may be less precise.

c. It is observed that when the follow-up period increases, the intervention found to increase the hemoglobin level.
