# Supplementary material for: Effect of Amaranth-Containing Dietary Intervention in Improving Hemoglobin Concentration: A Systematic Review and Meta-Analysis
Source: Public Health Rev. 2025 Jan 3;45:1607597. doi: 10.3389/phrs.2024.1607597 (PMC11738614; doi:10.3389/phrs.2024.1607597)
Supplement: Supplementary file 4 [file DataSheet2.docx]

**PubMed search strategy**

**Updated search strategy (****04/13/2024)**

The search was updated on April 13/2024 by repeating the initial search, then by the date of indexing to the database, and by adding the new keyword.

Effectiveness OR Efficacy

AND

Amaranth OR Amaranthus OR ((Amaranthus cruentus) OR (Amaranthus caudatus)) OR (Amaranthus hypochondriacus)

AND

(food) OR (Snack)

AND

Intervention

AND

((Hemoglobin) OR (Anemia)) OR (nutrition)

**The PubMed-generated search**

Search: ((((Effectiveness OR Efficacy) AND (Amaranth OR Amaranthus OR ((Amaranthus cruentus) OR (Amaranthus caudatus)) OR (Amaranthus hypochondriacus))) AND ((food) OR (Snack))) AND (Intervention)) AND (((Hemoglobin) OR (Anemia)) OR (nutrition)) AND (("2000/01/01"[Date - Entry]: "2024/04/13"[Date - Entry]))

("effect"[All Fields] OR "effecting"[All Fields] OR "effective"[All Fields] OR "effectively"[All Fields] OR "effectiveness"[All Fields] OR "effectivenesses"[All Fields] OR "effectives"[All Fields] OR "effectivities"[All Fields] OR "effectivity"[All Fields] OR "effects"[All Fields] OR ("efficacies"[All Fields] OR "efficacious"[All Fields] OR "efficaciously"[All Fields] OR "efficaciousness"[All Fields] OR "efficacy"[All Fields])) AND ("amaranth dye"[MeSH Terms] OR ("amaranth"[All Fields] AND "dye"[All Fields]) OR "amaranth dye"[All Fields] OR "amaranth"[All Fields] OR "amaranths"[All Fields] OR ("amaranthus"[MeSH Terms] OR "amaranthus"[All Fields]) OR ((("amaranthus"[MeSH Terms] OR "amaranthus"[All Fields]) AND "cruentus"[All Fields]) OR (("amaranthus"[MeSH Terms] OR "amaranthus"[All Fields]) AND ("caudate nucleus"[MeSH Terms] OR ("caudate"[All Fields] AND "nucleus"[All Fields]) OR "caudate nucleus"[All Fields] OR "caudatus"[All Fields]))) OR (("amaranthus"[MeSH Terms] OR "amaranthus"[All Fields]) AND "hypochondriacus"[All Fields])) AND ("food"[MeSH Terms] OR "food"[All Fields] OR ("snacked"[All Fields] OR "snacks"[MeSH Terms] OR "snacks"[All Fields] OR "snack"[All Fields] OR "snacking"[All Fields])) AND ("intervention s"[All Fields] OR "interventions"[All Fields] OR "interventive"[All Fields] OR "methods"[MeSH Terms] OR "methods"[All Fields] OR "intervention"[All Fields] OR "interventional"[All Fields]) AND ("haemoglobin"[All Fields] OR "hemoglobins"[MeSH Terms] OR "hemoglobins"[All Fields] OR "hemoglobin"[All Fields] OR "haemoglobins"[All Fields] OR "hemoglobin s"[All Fields] OR "hemoglobine"[All Fields] OR "hemoglobinization"[All Fields] OR "hemoglobinized"[All Fields] OR ("anaemia"[All Fields] OR "anemia"[MeSH Terms] OR "anemia"[All Fields] OR "anaemias"[All Fields] OR "anemias"[All Fields]) OR ("nutrition s"[All Fields] OR "nutritional status"[MeSH Terms] OR ("nutritional"[All Fields] AND "status"[All Fields]) OR "nutritional status"[All Fields] OR "nutrition"[All Fields] OR "nutritional sciences"[MeSH Terms] OR ("nutritional"[All Fields] AND "sciences"[All Fields]) OR "nutritional sciences"[All Fields] OR "nutritional"[All Fields] OR "nutritionals"[All Fields] OR "nutritions"[All Fields] OR "nutritive"[All Fields])) AND 2000/01/01:2024/04/13[Date - Entry]

Translations

Effectiveness: "effect"[All Fields] OR "effecting"[All Fields] OR "effective"[All Fields] OR "effectively"[All Fields] OR "effectiveness"[All Fields] OR "effectivenesses"[All Fields] OR "effectives"[All Fields] OR "effectivities"[All Fields] OR "effectivity"[All Fields] OR "effects"[All Fields]

Efficacy: "efficacies"[All Fields] OR "efficacious"[All Fields] OR "efficaciously"[All Fields] OR "efficaciousness"[All Fields] OR "efficacy"[All Fields]

Amaranth: "amaranth dye"[MeSH Terms] OR ("amaranth"[All Fields] AND "dye"[All Fields]) OR "amaranth dye"[All Fields] OR "amaranth"[All Fields] OR "amaranths"[All Fields]

Amaranthus: "amaranthus"[MeSH Terms] OR "amaranthus"[All Fields]

Amaranthus: "amaranthus"[MeSH Terms] OR "amaranthus"[All Fields]

Amaranthus: "amaranthus"[MeSH Terms] OR "amaranthus"[All Fields]

caudatus: "caudate nucleus"[MeSH Terms] OR ("caudate"[All Fields] AND "nucleus"[All Fields]) OR "caudate nucleus"[All Fields] OR "caudatus"[All Fields]

Amaranthus: "amaranthus"[MeSH Terms] OR "amaranthus"[All Fields]

food: "food"[MeSH Terms] OR "food"[All Fields]

Snack: "snacked"[All Fields] OR "snacks"[MeSH Terms] OR "snacks"[All Fields] OR "snack"[All Fields] OR "snacking"[All Fields]

Intervention: "intervention's"[All Fields] OR "interventions"[All Fields] OR "interventive"[All Fields] OR "methods"[MeSH Terms] OR "methods"[All Fields] OR "intervention"[All Fields] OR "interventional"[All Fields]

Hemoglobin: "haemoglobin"[All Fields] OR "hemoglobins"[MeSH Terms] OR "hemoglobins"[All Fields] OR "hemoglobin"[All Fields] OR "haemoglobins"[All Fields] OR "hemoglobin's"[All Fields] OR "hemoglobine"[All Fields] OR "hemoglobinization"[All Fields] OR "hemoglobinized"[All Fields]

Anemia: "anaemia"[All Fields] OR "anemia"[MeSH Terms] OR "anemia"[All Fields] OR "anaemias"[All Fields] OR "anemias"[All Fields]

nutrition: "nutrition's"[All Fields] OR "nutritional status"[MeSH Terms] OR ("nutritional"[All Fields] AND "status"[All Fields]) OR "nutritional status"[All Fields] OR "nutrition"[All Fields] OR "nutritional sciences"[MeSH Terms] OR ("nutritional"[All Fields] AND "sciences"[All Fields]) OR "nutritional sciences"[All Fields] OR "nutritional"[All Fields] OR "nutritionals"[All Fields] OR "nutritions"[All Fields] OR "nutritive"[All Fields]

**Initial search strategy keywords (10/30/2023)**

Effectiveness OR Efficacy

AND

Amaranth OR Amaranthus OR ((Amaranthus cruentus) OR (Amaranthus caudatus)) OR (Amaranthus hypochondriacus)

AND

(food) OR (Snack)

AND

Intervention

**The PubMed-generated search**

Search: **((((Effectiveness) OR (Efficacy)) AND (((((Amaranth) OR (Amaranthus)) OR (Amaranthus cruentus)) OR (Amaranthus caudatus)) OR (Amaranthus hypochondriacus))) AND ((food) OR (Snack))) AND (Intervention)** Filters: **from 2000 - 2023**

(("effect"[All Fields] OR "effecting"[All Fields] OR "effective"[All Fields] OR "effectively"[All Fields] OR "effectiveness"[All Fields] OR "effectivenesses"[All Fields] OR "effectives"[All Fields] OR "effectivities"[All Fields] OR "effectivity"[All Fields] OR "effects"[All Fields] OR ("efficacies"[All Fields] OR "efficacious"[All Fields] OR "efficaciously"[All Fields] OR "efficaciousness"[All Fields] OR "efficacy"[All Fields])) AND ("amaranth dye"[MeSH Terms] OR ("amaranth"[All Fields] AND "dye"[All Fields]) OR "amaranth dye"[All Fields] OR "amaranth"[All Fields] OR "amaranths"[All Fields] OR ("amaranthus"[MeSH Terms] OR "amaranthus"[All Fields]) OR (("amaranthus"[MeSH Terms] OR "amaranthus"[All Fields]) AND "cruentus"[All Fields]) OR (("amaranthus"[MeSH Terms] OR "amaranthus"[All Fields]) AND ("caudate nucleus"[MeSH Terms] OR ("caudate"[All Fields] AND "nucleus"[All Fields]) OR "caudate nucleus"[All Fields] OR "caudatus"[All Fields])) OR (("amaranthus"[MeSH Terms] OR "amaranthus"[All Fields]) AND "hypochondriacus"[All Fields])) AND ("food"[MeSH Terms] OR "food"[All Fields] OR ("snacked"[All Fields] OR "snacks"[MeSH Terms] OR "snacks"[All Fields] OR "snack"[All Fields] OR "snacking"[All Fields])) AND ("intervention s"[All Fields] OR "interventions"[All Fields] OR "interventive"[All Fields] OR "methods"[MeSH Terms] OR "methods"[All Fields] OR "intervention"[All Fields] OR "interventional"[All Fields])) AND (2000:2023[pdat])

**Translations**

**Effectiveness:** "effect"[All Fields] OR "effecting"[All Fields] OR "effective"[All Fields] OR "effectively"[All Fields] OR "effectiveness"[All Fields] OR "effectivenesses"[All Fields] OR "effectives"[All Fields] OR "effectivities"[All Fields] OR "effectivity"[All Fields] OR "effects"[All Fields]

**Efficacy:** "efficacies"[All Fields] OR "efficacious"[All Fields] OR "efficaciously"[All Fields] OR "efficaciousness"[All Fields] OR "efficacy"[All Fields]

**Amaranth:** "amaranth dye"[MeSH Terms] OR ("amaranth"[All Fields] AND "dye"[All Fields]) OR "amaranth dye"[All Fields] OR "amaranth"[All Fields] OR "amaranths"[All Fields]

**Amaranthus:** "amaranthus"[MeSH Terms] OR "amaranthus"[All Fields]

**Amaranthus:** "amaranthus"[MeSH Terms] OR "amaranthus"[All Fields]

**Amaranthus:** "amaranthus"[MeSH Terms] OR "amaranthus"[All Fields]

**caudatus:** "caudate nucleus"[MeSH Terms] OR ("caudate"[All Fields] AND "nucleus"[All Fields]) OR "caudate nucleus"[All Fields] OR "caudatus"[All Fields]

**Amaranthus:** "amaranthus"[MeSH Terms] OR "amaranthus"[All Fields]

**food:** "food"[MeSH Terms] OR "food"[All Fields]

**Snack:** "snacked"[All Fields] OR "snacks"[MeSH Terms] OR "snacks"[All Fields] OR "snack"[All Fields] OR "snacking"[All Fields]

**Intervention:** "intervention's"[All Fields] OR "interventions"[All Fields] OR "interventive"[All Fields] OR "methods"[MeSH Terms] OR "methods"[All Fields] OR "intervention"[All Fields] OR "interventional"[All Fields]
